# Superparamagnetic iron oxide nanoparticles regulate smooth muscle cell phenotype

**DOI:** 10.1002/jbm.a.35780

**Published:** 2016-05-30

**Authors:** Ioannis Angelopoulos, Paul Southern, Quentin A. Pankhurst, Richard M. Day

**Affiliations:** ^1^ Applied Biomedical Engineering GroupDivision of MedicineUniversity College LondonWC1E 6DDUK; ^2^ UCL Institute of Biomedical EngineeringUniversity College LondonLondonWC1E 6BTUK

**Keywords:** SPION, muscle, regenerative medicine, biomaterials

## Abstract

Superparamagnetic iron oxide nanoparticles (SPION) are used for an increasing range of biomedical applications, from imaging to mechanical actuation of cells and tissue. The aim of this study was to investigate the loading of smooth muscle cells (SMC) with SPION and to explore what effect this has on the phenotype of the cells. Adherent human SMC were loaded with ∼17 pg of unconjugated, negatively charged, 50 nm SPION. Clusters of the internalized SPION particles were held in discrete cytoplasmic vesicles. Internalized SPION did not cause any change in cell morphology, proliferation, metabolic activity, or staining pattern of actin and calponin, two of the muscle contractile proteins involved in force generation. However, internalized SPION inhibited the increased gene expression of actin and calponin normally observed when cells are incubated under differentiation conditions. The observed change in the control of gene expression of muscle contractile apparatus by SPION has not previously been described. This finding could offer novel approaches for regulating the phenotype of SMC and warrants further investigation. © 2016 Wiley Periodicals, Inc. J Biomed Mater Res Part A: 104A: 2412–2419, 2016.

## INTRODUCTION

Muscle insufficiency is a significant clinical challenge for a wide range of acquired and congenital clinical conditions. Improved therapies are sought that will increase muscle mass and restore organ function. Smooth muscle poses a specific set of challenges due to the lack of accessible sites suitable for harvesting autologous donor cells for regenerative medicine purposes. Smooth muscle cells (SMC) can switch between a differentiated “contractile” phenotype and a “proliferative” or “synthetic” phenotype.^1^, ^2^, ^3^, ^4^ The possibility of harnessing this phenotypic plasticity to form new tissues is an attractive strategy for the *ex vivo* bioengineering of various tissues, including arteries and sphincter muscle.^5^, ^6^ Shifting the proliferative SMC toward a contractile phenotype can be achieved via intra‐ or extracellular stimuli including soluble signalling factors, extracellular matrices, and mechanical stimulation. The resulting phenotypic state is characterized by the expression pattern of protein markers, proliferative capacity, and cell morphology.^7^, ^8^

SMC in the vasculature are subjected to continuous cyclic mechanical loading and the biological effects of this form of stimulation have been investigated extensively.^9^ Mechanical stimulation to control muscle phenotype has been achieved *in vitro* by culturing cells in a mechanically active environment, for example the Flexcell® Tension System, a computer‐regulated bioreactor that uses vacuum pressure to apply cyclic or static strain to cells cultured on flexible‐bottomed Bioflex culture plates. Using this system, deformation of the cytoskeleton has been shown to regulate cellular events and act as a potent mitogen, inducing proliferation of myoblasts and SMC *in vitro*.^9^, ^10^

Iron oxide nanoparticles are being increasingly used for a variety of biomedical applications. This includes imaging, targeted delivery of active pharmaceutical ingredients, tissue engineering, and destruction of tumour cells via hyperthermia.^11^ To date, their effect on smooth muscle phenotype has not been investigated. We have previously shown that negatively charged superparamagnetic iron oxide nanoparticles (SPION) are endocytosed by cells and held in discrete vesicles in the cytoplasm.^12^ Based on this observation we hypothesize that endocytosed clusters of SPION could have an effect on regulating cellular events, such as cell proliferation, differentiation, or morphogenesis. This could be particularly valuable for tissues such as muscle, where individual cells could be magnetically targeted to stimulate an increase in muscle cell growth and/or control of cell phenotype.

In the current study, we explore the concept of regulating cellular phenotype via loading of intracellular SPION using 50 nm negatively charged SPION.

## MATERIALS AND METHODS

### Cell culture

Primary cultures of human rectal smooth cells (HRSMC) were obtained from ScienCell^TM^ (Catalogue Number: 2980). Cells were cultured in either proliferation medium consisting of Minimum Essential Medium Eagle (Sigma Aldrich, UK) supplemented with 10% foetal bovine serum (FBS) (Life Technologies Ltd, UK), 1× non‐essential amino acids (NEAA), 2 m*M* glutamine, 50 U/mL penicillin, and 50 µg/mL streptomycin (Sigma Aldrich, UK), or differentiation medium consisting of Dulbecco's Modified Eagle Medium (Sigma Aldrich, UK) supplemented with 1× NEAA, 2 m*M* glutamine, 50 U/mL penicillin, 50 µg/mL streptomycin, and 2 ng/mL transforming growth factor (TGF)‐β (PeproTech EC Ltd, UK).

### Loading of SPION in HRSMC

Unconjugated, negatively charged SPION (fluidMAG‐UC/A; Chemicell GmbH, Berlin, Germany) was used for all experiments. This consisted of an aqueous dispersion with a stock concentration of 25 mg/mL and particle density of ∼1.3 × 10^16^ particles/g. The SPION were uncoated and had an anionic surface charge. The particle size, determined by the manufacturer using photon correlation spectroscopy, was 50 nm, which corresponds to the hydrodynamic diameter of the multi‐core domain structures consisting of a cluster of several 8–15 nm single domain iron oxide crystals and associated hydrogen‐bonded shell of water molecules.

HRSMC grown in 75‐cm^2^ tissue culture flasks were incubated at 37°C and 5% CO_2_ in proliferation medium supplemented with SPION at a final concentration of 250 µg/mL. After 24 h, the cells were washed five times with 10 mL of phosphate buffered saline (PBS), were detached by trypsinization, and re‐seeded for a further 24 h. Then the culture medium was replaced with proliferation or differentiation medium for 7 days.

### Quantification of SPION in HRSMC

Cells incubated with SPION were washed and detached by trypsinization followed by washing and centrifugation. After performing a cell count, cells were centrifuged again and the pellet lyophilized overnight. The amount of SPION loaded into the cells was measured by superconducting quantum interference device (SQUID) magnetometry. A Quantum Design SQUID‐VSM magnetometer (Quantum Design Inc, San Diego, CA) was used to apply a magnetic field to each sample in the range of 7 T to −7 T at a temperature of 300 K. A background diamagnetic component from the sample holder and diamagnetic compounds in the sample was determined from the linear regions of the graph (at fields above +3T and below −3T) and removed. The saturation magnetic moment due to the SPION in the samples thus obtained was used to estimate the SPION mass per cell, assuming a saturation magnetization for the SPION of 73 emu/g. This was then plotted against the concentration of SPION in the incubation medium.

### Ultrastructural localization of SPION

Transmission electron microscopy (TEM) was used to determine the cellular localization of SPION in HRSMC attached to the base of the tissue culture plates. After loading and washing, samples were fixed in 2% paraformaldehyde + 2.5% gluteraldehyde in 0.1*M* cacodylate buffer and subsequently post‐fixed in a solution of phosphate buffered 1% osmium tetroxide for 30 min to avoid any contrast masking on the SPION. After progressive dehydration series in ethanol, the base of the plates were scored with a scalpel and the monolayer of cells were detached from the base of the plate by adding propylene oxide. The cell sheets were placed in two further changes of propylene oxide to remove residual tissue culture plastic from the cells and to act as a transition solvent before the embedding stage. In order to enhance the infiltration of samples with an Araldite CY212 resin (Agar Scientific), two further stages of 90 min each in mixtures of 1:3 and 1:1 resin/propylene oxide at room temperature were allowed, after which the cells were embedded in pure Araldite medium at 60°C for 36 h. The resulting blocks were thin sectioned in an ultramicrotome and directly collected onto copper grids for examination under a JEOL JEM 1200EX TEM operating at 80 kV.

Scanning electron microscopy (SEM) was used to investigate surface features of HRSMC incubated with SPION. Cells were fixed and dehydrated, as described above before being mounted on an aluminium stub using silver paint. Samples were coated with gold/palladium before examination under a JSM‐7500F scanning electron microscope (JEOL, USA).

### Immunocytochemistry

The expression of smooth muscle contractile proteins using monoclonal antibodies specific to α‐smooth muscle actin (clone 1A4, 1:1000; Sigma), calponin (clone hCP, 1:1000; Sigma), caldesmon (clone hHCD, 1:500; Sigma), and myosin heavy chain (clone hSM‐V, 1:500; Sigma) was investigated by immunocytochemistry. HRSMC were seeded in 35‐mm Fluorodish^TM^ cell culture dishes (World Precision Instruments, USA) and incubated as specified for each experiment. The medium was removed and cells were fixed in 4% formaldehyde in PBS for 10 min at room temperature. After washing in PBS, cells were incubated in 3% BSA + 0.1% Triton in PBS for 1 h at room temperature to block non‐specific protein binding and to permeabilize the cells. The primary antibody diluted in PBS was added to each dish and incubated for 1 h at room temperature, followed by washing and incubation for 1 h with anti‐mouse IgG FITC conjugated secondary antibody (F9006, 1:500; Sigma). The dishes were mounted with glass coverslips using mounting medium containing DAPI (Vectashield® Mounting Medium with DAPI, Vector Laboratories Ltd, UK). Images were acquired using a fluorescence microscope (BX51 Olympus, GX Optical, UK).

### Expression of smooth muscle cell genes

RNA was extracted from cells using the RNeasy mini kit (Qiagen, UK), following manufacturer's instructions. The amount and quality of RNA in each sample was quantified using a TA NanoDrop ND‐1000 spectrophotometer (Thermoscientific, UK). Complementary DNA (cDNA) libraries were prepared using a cDNA synthesis kit (Applied Biosystems, UK). Real time polymer chain reaction (PCR) was used to quantify the amount of cDNA present in the sample. The expressions of actin (ACTA2), myosin heavy chain (MYH11), caldesmon CALD1, and calponnin (CNN1) were investigated, along with GAPDH as the house keeping gene, using commercially available primers (Applied Biosystems, UK). The PCR reaction mixtures were transferred to a 384‐well plate (MicroAmp^TM,^ Applied Biosystems) and sealed with a film lid. The plate was centrifuged and placed in a LightCycler 480 (Roche, UK) for the real time PCR reaction to occur. The sample reading was normalized according to the housekeeping gene GAPDH.

### Cell proliferation and metabolic assays

The proliferation of HRSMC was measured using a Colorimetric BrdU ELISA Assay (Roche, UK) following the manufacturer's instructions. The metabolic activity of HRSMC cells was measured using the CellTiter 96 Aqueous Non‐Radioactive Cell Proliferation Assay (Promega, UK).

### Data analysis

Data analysis was performed using GraphPad Prism version 5.00 for Windows, (GraphPad Software, San Diego, CA). All experiments were repeated in replicates of four, except for the PCR analysis which was repeated in triplicate. Statistical significance was tested using one‐way ANOVA and Tukey test *post hoc* analysis. Values were considered significant when *p* < 0.05.

## RESULTS

### Loading HRSMC with SPION

**Figure 1:**
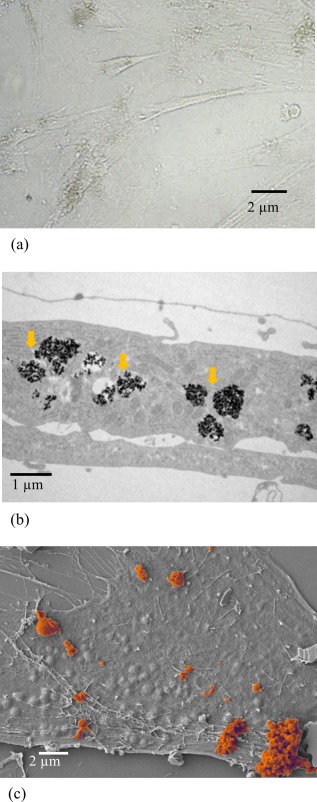
(a) Light microscopy of HRSMC following incubation with culture medium containing 250 µg/mL SPION for 24 h. SPION is visible as brown material in the cells. (b) Transmission electron microscopy of HRSMC following incubation with culture medium containing 250 µg/mL SPION for 24 h. SPION particles are internalized in vesicles (arrows) inside the cells. (c) Scanning electron microscopy of SPION clusters (pseudo colored brown) attached to the surface of HRSMC following incubation with culture medium containing 250 µg/mL SPION for 24 h.

Following incubation of HRSMC in proliferation medium containing 250 μg/mL SPION for 24 h and subsequent washing stages to remove unbound particles, SPION clusters associated with the cells were clearly visible using light microscopy [Figure 1(a)]. SPION loaded cells retained the spindle morphology similar to that seen with control cells. TEM of the HRSMC incubated with SPION revealed the particles were held in vesicles inside the cytoplasm [Figure 1(b)]. SEM indicated SPION clusters on the cell surface despite extensive washing stages, suggesting the particles were tethered to the surface [Figure 1(c)].

### Effect of switching between proliferation and differentiation medium following loading HRSMC with SPION

**Figure 2:**
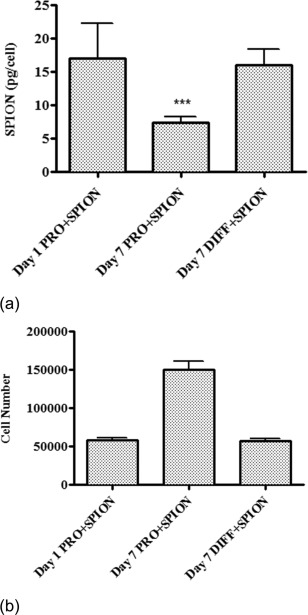
(a) SQUID measurement of the amount of iron loaded per cell after incubation of HRSMC in culture medium containing 250 µg/mL SPION for 24 h (day 1 PRO + SPION), or incubation for a further 7 days in proliferation medium (day 7 PRO + SPION) or differentiation medium (day 7 DIFF + SPION). The amount of iron loaded per cell was significantly lower after 7 days incubation in proliferation medium compared with amount measured at day 1. (b) Cell counts of HRSMC after incubation in culture medium containing 250 µg/mL SPION for 24 h (day 1 PRO + SPION), or incubation for a further 7 days in proliferation medium (day 7 PRO + SPION) or differentiation medium (day 7 DIFF + SPION). (***p < 0.001).

SQUID magnetometry was used to quantify the amount of SPION loading in HRSMC after incubation in proliferation medium for 24 h. The average remaining amount of SPION per cell after 7 days incubation in proliferation medium was reduced compared with the average amount per cell measured after 24 h incubation (Figure 2). The amount was also reduced compared with the average amount of SPION measured in cells incubated in differentiation medium for 7 days. This coincided with an increase in cell number after 7 days incubation in proliferation and no change in cell number for cells incubated in differentiation medium for 7 days.

**Figure 3:**
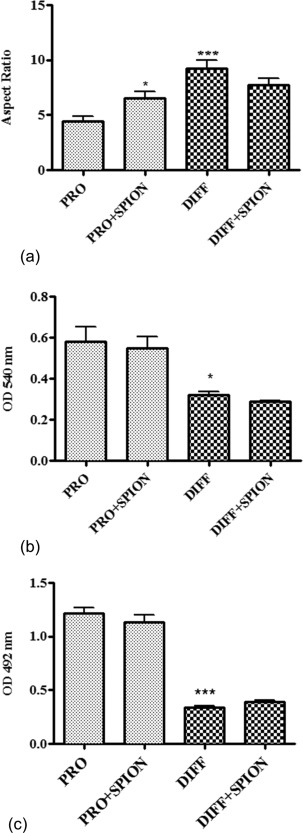
The effect of switching HRSMC from incubation in proliferation medium to differentiation medium for 7 days. (a) Cell aspect ratio, (b) BrdU ELISA cell proliferation assay, and (c) cell metabolic assay measured using the CellTiter 96 aqueous non‐radioactive cell proliferation assay. (*p < 0.05, ***p < 0.001).

The aspect ratio increased from 4.46 ± 0.44 for cells incubated in proliferation medium to 9.23 ± 0.71 for cells incubated in differentiation medium (*p* < 0.001). Loading cells with SPION also caused a small increase in the aspect ratio to 6.54 ± 0.64 for cells incubated in proliferation medium and decreased to 7.77 ± 0.58 for cells incubated in differentiation medium [Figure 3(a)].

Incubation of HRSMC in differentiation medium caused a significant reduction in cell proliferation compared with cells incubated in proliferation for 7 days [Figure 3(b)]. However, loading cells with SPION before incubation in proliferation or differentiation medium did not cause any further change for either group. An identical pattern was observed with the measurements of cell metabolic activity [Figure 3(c)].

**Figure 4:**
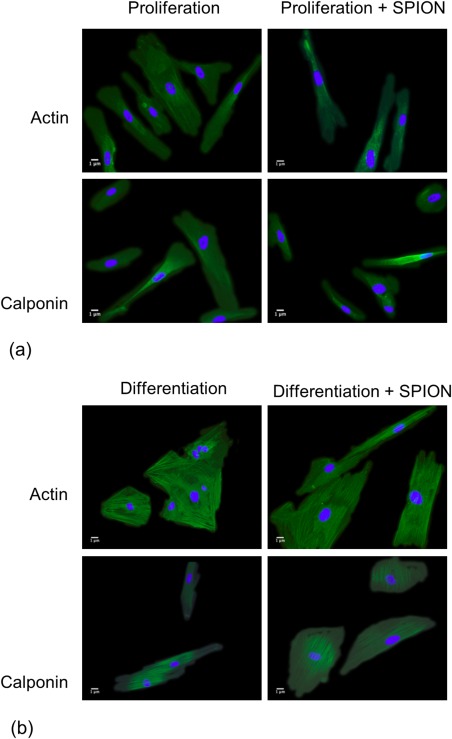
Fluorescence microscopy of HRSMC stained for the contractile proteins actin and calponin. (a) After incubation in proliferation medium ± 250 µg/mL SPION for 24 h followed by incubation in proliferation medium for 7 days. (b) After incubation in proliferation medium ± 250 µg/mL SPION for 24 h followed by incubation in differentiation medium for 7 days.

The effect that loading cells with SPION had on the differentiation of HRSMC toward a contractile phenotype was investigated by switching from proliferation to differentiation medium. Cells were initially incubated in proliferation medium containing 250 μg/mL SPION for 24 h, followed by washing stages to remove unbound particles. At this stage, the cells were incubated with either proliferation medium or differentiation medium for a further 7 days. Strong actin and calponin staining was visible in cells incubated in proliferation medium. The staining was mainly diffuse and distributed throughout the cytoplasm. The loading of SPION did not change this staining pattern (Figure 4). The staining pattern of actin and calponin changed following incubation in differentiation medium for 7 days. Staining remained strong for both proteins but exhibited a filamentous distribution. No staining was visible for caldesmon or myosin heavy chain for any of the groups.

**Figure 5:**
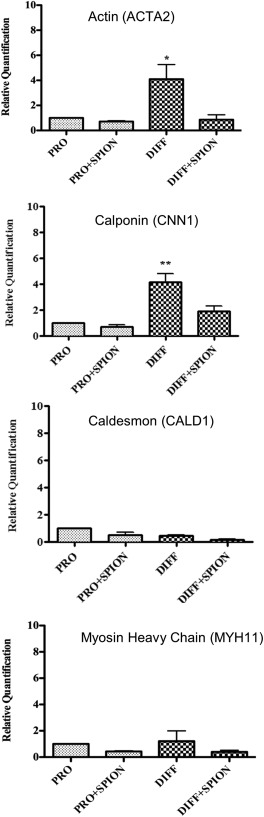
Gene expression of the actin, calponin, caldesmon, and myosin heavy chain in HRSMC after incubation in proliferation medium ± 250 µg/mL SPION for 24 h followed by incubation in proliferation medium or differentiation medium for 7 days. Quantification is relative to the expression of each marker following incubation in proliferation medium alone for 7 days, which was normalized to a value of 1. (*p < 0.05; **p < 0.01).

The relative gene expression of actin and calponin were both increased fourfold following incubation in differentiation medium for 7 days compared with cells incubated in proliferation medium for the same period of time (Figure 5). Incubation of HRSMC with SPION for 24 h before incubation in differentiation medium for 7 days inhibited the increase in actin and calponin gene expression. Loading HRSMC with SPION followed by incubation in proliferation medium for 7 days produced no change in the relative gene expression. No increase in the expression of caldesmon or myosin heavy chain was measured for any of the groups.

## DISCUSSION

The aim of the current study was to investigate what effect the loading of SMC with SPION would have on the cell phenotype. Incubation of HRSMC with negatively charged, uncoated SPION added to the tissue culture medium for 24 h led to the loading of cells with magnetic material. Magnetometry data indicate SPION loading by HRSMC was not significantly increased by increasing the concentration of SPION in the culture medium (data not shown). Following washing stages to remove unbound SPION from the cells, TEM indicated much of the SPION was internalized and held in endosome‐like structures. SEM of the cell surface indicated clusters of SPION also remained tethered to the cell. It is possible these clusters could be in the process of undergoing either endocytosis or exocytosis. TEM revealed internalized SPION was localized to vesicles that had been transported into the cytoplasm.

Previous studies have performed detailed investigations of the uptake of SPION by cells.^13^, ^14^ Use of chemical inhibitors in these studies indicates uptake via endocytosis of SPION occurs via a scavenger receptor pathway independent of dynamin, caveolae, and clathrin‐coated pit. Although these studies were conducted with different SPION and cell types, they provide an indication of the type of specific uptake pathways likely to be occurring in the current study.

Appropriate spatial and temporal control of cellular apparatus at the gene and protein level is essential for SMC to retain the correct phenotype according to the functional requirements of their native tissue. In tissues such as the aorta there is a mixture of SMC with proliferative and contractile phenotypes.^15^ This differs from the *in vitro* expansion of SMC in culture, where there is a predominance of cells in a proliferative phenotype and suppression of protein expression associated with the contractile cell phenotype. The relative expression of contractile apparatus, such as α‐smooth muscle actin, calponin, caldesmon, and myosin heavy chains can be used to characterize SMC cells along the proliferative‐contractile continuum.^16^, ^17^ α‐Smooth muscle actin and calponin are considered to be early and intermediate markers of smooth muscle cell differentiation, whereas caldesmon and myosin heavy chains are intermediate to late markers of differentiation.^18^

HRSMC in the current study were shifted toward a more contractile, less proliferative phenotype by incubating cells in FCS depleted culture medium supplemented with TGF‐β1. HRSMC initially incubated in proliferation medium exhibited diffuse cytoplasmic staining of α‐smooth muscle actin and calponin. When the cells were switched to differentiation medium for 7 days, the localization of αSMA and calponin staining became filamentous. Similarly, the switch to differentiation medium resulted in a fourfold increase in the gene expression of α‐smooth muscle actin and calponin. These observations are in keeping with previous studies that have shown TGFβ1 induces the expression and organization of contractile apparatus in SMC.^19^, ^20^, ^21^, ^22^, ^23^ Further evidence of HRSMC differentiation toward a contractile, less proliferative phenotype was the decrease in cell proliferation compared with cells incubated for the same length of time in proliferation medium, and an increase in the cell aspect ratio. Interestingly, the average amount of SPION per cell decreased after 7 days incubation in proliferation medium compared with the starting amount, whereas the amount remained unchanged for cells incubated in differentiation medium. This is likely to result from the internalized SPION being divided amongst progeny as the cells proliferated. Accordingly, the cells in differentiation medium did not proliferate, so the average amount of SPION per cell remained unchanged. Neither caldesmon nor myosin heavy chain protein was detected in HRSMC incubated in proliferation or differentiation medium. The reason for this might relate to their temporal expression and the duration of incubation in differentiation medium performed in the current experiments.

Incubation of HRSMC in proliferation or differentiation medium containing SPION did not affect the intensity or localization of protein staining observed in the cells. However, loading HRSMC with SPION inhibited the gene expression of α‐smooth muscle actin and calponin when cells were switched to differentiation medium compared with unloaded HRSMC. The mechanism underlying the inhibition of these two genes, both of which are associated with the shift of SMC from a proliferative to contractile phenotype, is uncertain at this stage, but could be related to interference with TGFβ1 signaling by inhibition of SMAD genes. SMC differentiation via TGF‐β1 binding occurs via signalling in the SMAD pathway, causing inhibition of cell proliferation and induction of contractile proteins in SMCs.^8^, ^20^, ^21^, ^24^, ^25^ Gene expression profiling of HeLa cells following incubation with 26 nm magnetite nanoparticles and their internalization resulted in down‐regulation of 68 genes, many of which were involved in the TGFβ signaling pathway.^26^ Although the effects of SPION on the regulation of gene expression are likely to be cell specific, this evidence implicates interference of TGFβ and SMAD signal transduction. The protein products of actin and calponin play a role in force generation and modulation of myosin ATP‐ase activity, respectively.^27^, ^28^ Future studies will focus on the down‐stream events of gene regulation produced by SPION, including incubation periods that extend beyond 7 days and cell contraction studies to provide a more robust indication of SMC phenotype and to clarify whether these observed effects result in changes to the functional capacity of HRSMC. In addition, another line for future study will be to investigate the possible mechanical effects associated with the SPION uptake by the cells. It is notable that at about 17 pg, the measured SPION mass per cell is a sizeable fraction of the estimated about 30 pg mass of an adherent HRSMC (assuming 5 μm diameter, 1.5 μm thickness, 1 g/cc density), which might in itself have a significant effect on the characteristics of the contractile phenotype of the cell. It would be very interesting to explore this further, as a function of the SPION mass per cell loading.

In conclusion, SPION internalized by HRSMC prevented the up‐regulation of calponin and actin mRNA normally observed in response to incubation with TGF‐β1. This might offer novel therapeutic opportunities for the use of SPION in regenerative medicine relating to control of the muscle phenotypic continuum. It will be important to understand the consequence of these changes on the function of SMC, particularly whether this effect is specific to HRSMC, and whether similar effects occur to SMC at other stages of the phenotypic continuum *in vitro* and *in vivo*.
